# Recent advances in cerebral oximetry. Assessment of cerebral autoregulation with near-infrared spectroscopy: myth or reality?

**DOI:** 10.12688/f1000research.11351.1

**Published:** 2017-08-31

**Authors:** Anneliese Moerman, Stefan De Hert

**Affiliations:** 1 Department of Anesthesiology, Ghent University Hospital, Ghent, Belgium

**Keywords:** Cerebral autoregulation, NIRS, Oximetry

## Abstract

In recent years, the feasibility of near-infrared spectroscopy to continuously assess cerebral autoregulation has gained increasing interest. By plotting cerebral oxygen saturation over blood pressure, clinicians can generate an index of autoregulation: the cerebral oximetry index (COx). Successful integration of this monitoring ability in daily critical care may allow clinicians to tailor blood pressure management to the individual patient’s need and might prove to be a major step forward in terms of patient outcome.

Near-infrared spectroscopy (NIRS) has been adopted in clinical practice for over three decades now. Based on a similar principle as pulse oximetry, it allows continuous, non-invasive, real-time monitoring of cerebral oxygen saturation (S _c_O _2_) in a small sample of the frontal cortex. It was initially promoted as a brain monitor in cardiac surgery and neonatal intensive care, but its use has been extended to various non-cardiac surgeries and critical care settings. Some recent reviews provide excellent detail on the principles of NIRS and its current clinical practice patterns ^1– 3^.

The present review focuses on an emerging aspect of monitoring abilities with NIRS, which, when combined with continuous blood pressure monitoring, might prove to be a major step forward in critical care management: cerebral autoregulation monitoring.

## You cannot manage what you do not measure

**Figure 1.**
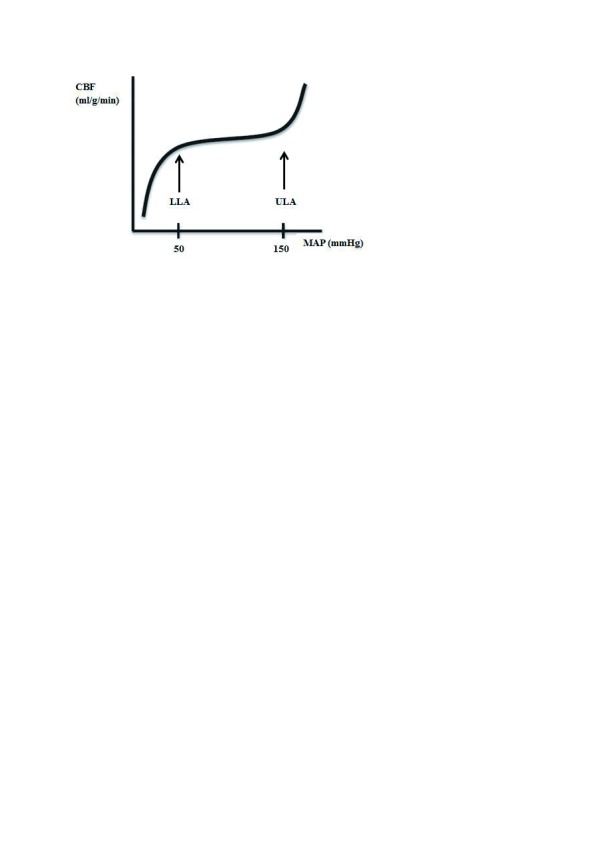
Classic depiction of cerebral autoregulation, with autoregulation plateau, lower limit of autoregulation (LLA), and upper limit of autoregulation (ULA). CBF, cerebral blood flow; MAP, mean arterial blood pressure.

Currently, blood pressure targets during perioperative and critical care management are mostly empirically chosen and rather fixed in any individual patient. This practice finds its origin in the concept of cerebral autoregulation, which refers to a modulating mechanism that controls cerebral blood flow (CBF) during changes in cerebral perfusion pressure (CPP). The classic curve of cerebral autoregulation represents the autoregulation plateau and the lower and upper limit of autoregulation (LLA and ULA, respectively) ( Figure 1).

Using data from 11 human studies, derived from young patients with few comorbidities, Lassen described an autoregulatory plateau between mean arterial blood pressures (MAPs) of 50 and 150 mm Hg, over which CBF remained constant by active myogenic control of small cerebral arteries and arterioles ^4^. At the extremes of blood pressure (that is, below the LLA and above the ULA), myogenic vasoactivity capacity is no longer able to accommodate these changes, and CBF becomes pressure-passive; CBF decreases when MAP moves below the LLA and increases when MAP is above the ULA.

**Figure 2.**
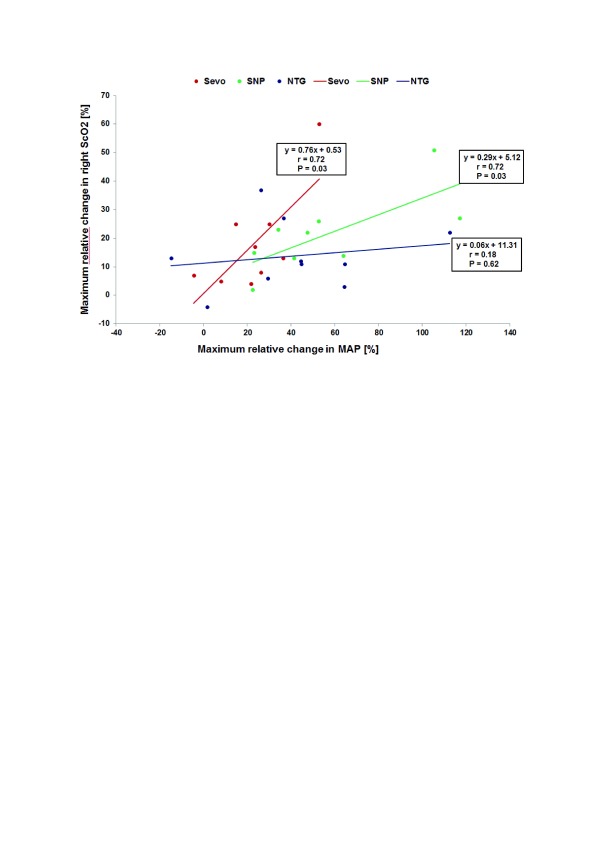
Correlation of changes in mean arterial pressure (MAP) with changes in cerebral oxygen saturation (S _c_O _2_) with administration of three different vasodilating drugs. The drugs are sevoflurane (Sevo), sodium nitroprusside (SNP), and nitroglycerin (NTG). Own data, not published.

This description of cerebral autoregulation has been widely accepted and has been propagated in many textbooks, and, even today, most caregivers feel confident with a MAP within the presumed autoregulation range. However, already in 1997, the validity of this concept was criticized ^5^. It is now accepted that CBF regulation is determined by many more factors than blood pressure alone. A variety of physiological and biochemical mechanisms may interact and lead to a complex entity of vascular reactivity in which the relative regulatory role of each component remains largely unknown ^6, 7^. It has also been acknowledged that autoregulation may be altered in specific diseases (for example, prematurity, hypertension, diabetes, and stroke) and in specific conditions (for example, changes in carbon dioxide and pharmacologic interference) ^6– 12^. Figure 2 gives an example from one of our own studies ^13^. In this study, we managed hypertension with one of three different vasodilating drugs: sevoflurane, sodium nitroprusside (SNP), or nitroglycerin. Figure 2 clearly shows that, with sevoflurane and SNP, changes in MAP are highly correlated with changes in S _c_O _2_, suggesting impairment of cerebral autoregulation.

**Figure 3.**
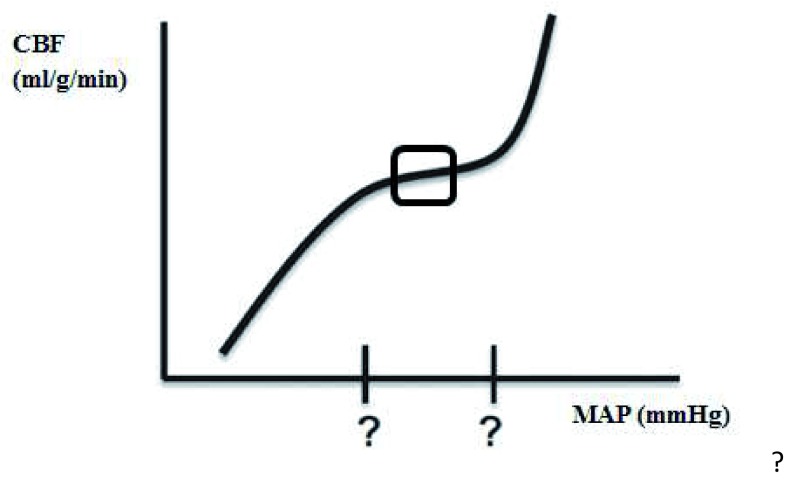
The autoregulation curve may vary from the classic depiction of autoregulation, with unknown limits of autoregulation (question marks), and an optimal mean arterial blood pressure (square). CBF, cerebral blood flow; MAP, mean arterial blood pressure.

In response to all of these confounders affecting cerebral vascular reactivity, the limits of autoregulation and the shape of the autoregulation curve may vary enormously and unpredictably ( Figure 3) ^14, 15^. Therefore, the practice of applying a fixed blood pressure threshold in an individual patient is risky, and tighter pressure control is mandatory ^16^.

Even with an impaired autoregulation curve, there might be an optimal blood pressure in the middle of the autoregulation curve at which the autoregulatory function is most robust ( Figure 3, indicated with a square). Identifying the range of optimal cerebral autoregulation offers the potential for individualization of blood pressure targets, and integrating the ‘optimal MAP’ in daily critical care management might shift the current clinical paradigm of ‘one size fits all’ care to individualized, patient-specific, physiology-based blood pressure management.

## Can near-infrared spectroscopy be used to assess cerebral autoregulation?

To assess cerebral autoregulation, changes in CBF are plotted over a wide range of blood pressures. From a practical point of view, this requires a continuous measurement of arterial blood pressure (ABP) and a real-time estimate of CBF. Although theoretically CPP should be plotted, this requires invasive monitoring of intracranial pressure (ICP) (CPP = MAP − ICP). Therefore, without access to ICP monitoring and without an increased ICP, ABP is considered a valid substitute for CPP.

For the assessment of CBF, no convenient means of measuring CBF currently exist, so surrogates of CBF are being used. Recently, the various indices of autoregulatory function were extensively examined in adults with traumatic brain injury (TBI) ^17^.

**Figure 4.**
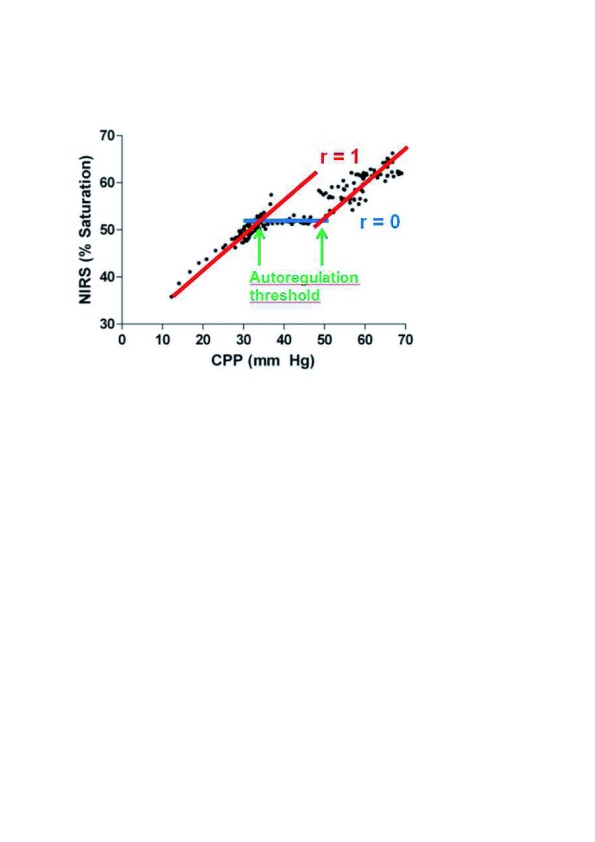
Near-infrared spectroscopy (NIRS)-derived oxygen saturation versus cerebral perfusion pressure (CPP), depicting the cerebral autoregulation curve. The correlations generate the cerebral oximetry index (COx). Adapted from Brady *et al*. ^18^.

NIRS-measured S _c_O _2_ is considered a reliable surrogate of CBF. When S _c_O _2_ is correlated with MAP, an index of autoregulatory vasoreactivity, the cerebral oximetry index (COx), is generated. Blood pressure in the autoregulation range is indicated by a COx that approaches zero; that is, there is no correlation between S _c_O _2_ and MAP. A COx approaching one indicates a strong correlation between the two signals and has to be interpreted either as impaired autoregulation or as MAP being beyond the limits of autoregulation ( Figure 4). The COx has been validated and had good agreement with transcranial Doppler-derived measurements of pressure autoregulation in piglets ^18^ and in adult patients ^19– 21^.

## Current clinical applications of cerebral autoregulation monitoring with near-infrared spectroscopy

Studies which evaluate cerebral autoregulation with the use of NIRS have been expanding over the past few years. Owing to the high incidence of brain injury in these domains, research on this topic has been established mainly in the areas of neonatology, cardiac surgery, and neurocritical care.

NIRS was originally introduced in clinical practice in 1985 for the assessment of cerebral oxygenation in preterm infants ^22^. Organ immaturity makes neonates vulnerable to physiological disturbances, and especially the brain may be at greater risk because of the incompletely developed cerebral autoregulation mechanism ^23, 24^. The need for firm hemodynamic boundaries has challenged continuous research in this area. We refer the interested reader to some recent reviews on this topic ^9, 25, 26^.

In cardiac surgery patients, an ABP of 50–60 mmHg is widely considered to be an acceptable perfusion pressure during cardiopulmonary bypass (CPB). However, this practice was challenged by data showing a lower incidence of cardiac and neurologic complications when targeting a higher MAP (>80 mmHg) during CPB ^27^. Impaired cerebral autoregulation has been demonstrated in 35% of cardiac surgery patients ^12^, with a wide variability of the MAP at the LLA from 40 to 90 mmHg ^14^. Consequently, in the case of empirically chosen MAP targets, patients may spend varying portions of time with MAP below the LLA during CPB.

In some recent studies, it has been demonstrated that MAP variations outside the autoregulatory range, not the absolute MAP values, were associated with adverse outcomes ^28– 31^. The time and magnitude that MAP spent below the LLA were higher for patients who developed acute kidney injury compared with patients without acute kidney injury, although absolute blood pressure values were equal between the two groups ^28^. A significant association was also found between blood pressure excursions below the LLA and major morbidity (for example, renal failure, duration of mechanical lung ventilation, and low cardiac output syndrome) ^29^. A recent study demonstrated that a blood pressure below the optimal MAP based on COx monitoring was associated with an increase in postoperative plasma GFAP (glial fibrillary acidic protein), which is a brain-specific injury biomarker ^30^. However, on the other hand, simply raising blood pressure might result in a MAP above the ULA, which also predisposes to cerebral injury. Hori *et al*. demonstrated that the sum of the product of the magnitude and duration of MAP above the ULA was associated with increased risk for delirium in cardiac surgery patients ^31^.

Patients with brain injuries constitute another population at high risk of impaired autoregulation. A consensus statement from the Neurocritical Care Society and the European Society of Intensive Care Medicine suggested that continuous monitoring of cerebral autoregulation might help guide ABP and CPP targets to ‘optimal’ levels in patients with TBI and might contribute to prognostication ^32^. Management at or close to the optimal CPP has been shown to be associated with better outcomes in patients with TBI ^33, 34^. Since an in-depth discussion of cerebrovascular autoregulation monitoring in neurocritical care is beyond the scope of this article, we refer the interested reader to some excellent reviews ^6, 35– 39^.

One methodological aspect of autoregulation monitoring with NIRS in the setting of neurosurgery and neurointensive care requires close attention. NIRS technology is based on sending near-infrared light through the tissues, where it is attenuated because of a combination of absorption and scattering. It is a prerequisite that the quantity of light scattering remains constant during the measurements and that changes in attenuation result solely from changes in absorption ^40^. However, in conditions of brain injury, tissue composition may vary substantially (contusions, hemorrhages, and brain swelling), resulting in changes in light absorption and scattering. Accurate quantification of S _c_O _2_ and data quality could be questioned in this setting ^41^.

## Barriers to assessing cerebral autoregulation with near-infrared spectroscopy

While at first view defining COx might seem simple and easy to perform at the bedside, some major barriers have to be faced.

### Barrier 1: is cerebral oxygen saturation a reliable surrogate of cerebral blood flow?

The use of NIRS for quantifying cerebral autoregulation is based on some assumptions, and this continues to be a major point of discussion. S _c_O _2_ is determined by arterial blood oxygen content, cerebral oxygen consumption, oxygen-tissue diffusivity, and CBF. It has been assumed that, if all other parameters which influence S _c_O _2_ are kept constant, changes in S _c_O _2_ are due to changes in CBF and therefore that those two parameters can be interchanged. Although one might question this approach, the problem remains that to date there is no continuous real-time methodology to quantify CBF.

### Barrier 2: autoregulation data analysis

In most studies, COx is determined by using ICM+ ^®^ software (Cambridge Enterprise Ltd, Cambridge, UK, http://www.neurosurg.cam.ac.uk/icmplus). The S _c_O _2_ signal and the MAP signal are captured longitudinally (time-domain analysis). Filtering of both signals is performed to limit analysis to the frequency of slow waves (0.05–0.003 Hz), which are relevant to autoregulation and exclude confounding wave components such as respiratory and pulse frequencies. A sliding analysis window of a 300-second period updated every 10 seconds runs across the signals, and the S _c_O _2_ and MAP data within these windows are plotted against each other, incorporating 30 data points for each calculation. The linear regression line for these data is computed, and the Pearson correlation coefficient is obtained. The process repeats as the analysis window scans over the signals in incremental steps, resulting in a continuous COx measurement.

To calculate the optimal MAP, the COx values are binned in 5 mmHg blood pressure increments. The first and last MAP bins, as well as bins which contain less than 2% of data points, are discarded. ICM+ ^®^ then fits a U-shaped curve through the COx values plotted versus MAP. The optimal MAP can be identified at the point with the lowest COx.

However, owing to the granular nature imposed on the bin-aggregated data, binning makes it difficult to produce a robust automated algorithm to assess autoregulation ^42^. The binned data are generally noisy in a clinical environment, which may be device-specific (for example, arterial line flushing or poor NIRS signal) or physiological in nature (for example, caused by administration of drugs or positional changes). This becomes even more important when very few bins contain data points, such as early in a procedure when the complete picture of the COx plot has not yet built up, or when blood pressure is relatively stable. Montgomery *et al*. demonstrated that the data in their raw format (that is, unbinned) are feasible for monitoring cerebral autoregulation, and they hypothesized that their method might be particularly useful in noisy environments ^42^.

The current methodology for analysis of cerebral autoregulation includes either a time-domain or a frequency-based approach. The most common method is to calculate the correlation coefficient between S _c_O _2_ and MAP (time-domain approach). However, it could be argued that cerebral autoregulation is a complex physiological system and that simple correlation analysis does not cope with the complex interplay and the time-varying aspects of the different physiological mechanisms ^43^. Coherence and transfer function analyses have also been used to quantify cerebral autoregulation ^44, 45^. Caicedo *et al*. analyzed four different measurement models used for cerebral autoregulation assessment (correlation, coherence, modified coherence, and transfer function). Although Caicedo *et al*. proposed transfer function gain as the most robust method when used for cerebral autoregulation studies, correlation was also considered a robust method, despite some restrictions related to time delay ^46^.

### Barrier 3: autoregulation data interpretation

Varying definitions to decide between intact and impaired autoregulation are found in the literature. Generally, a COx of less than 0.3 is considered consistent with intact autoregulation and the MAP with the lowest COx is considered the optimal MAP. However, noise on the data may impede the identification of intact and impaired regions. A further complication for the assessment of autoregulation is that, owing to interpatient variability, no specific threshold will apply to everyone. Moreover, it has been postulated that the flat part of the autoregulation curve is never really horizontal, but slightly tilted ^47^. So autoregulation is not an on–off phenomenon, in which correlations between CBF and MAP jump from zero (intact autoregulation) to one (impaired autoregulation), and this makes the interpretation of autoregulation data rather cumbersome.

### Barrier 4: assessment of the autoregulation phenomenon

One approach to assess cerebral autoregulation is to monitor the S _c_O _2_ response to natural slow variations in blood pressure. However, the natural blood pressure variations may not be strong enough to challenge CBF. An alternative approach is to induce blood pressure variations (for example, by vasoactive drugs ^12^, change in body position ^48^, or release of a thigh cuff ^49^) and measure the concomitant response of S _c_O _2_. However, it has been demonstrated that CBF dynamics are driven by the rate of change in blood pressure rather than absolute pressure per se; therefore, the precipitous change in pressure might elicit autoregulatory reactions that would not be seen with gradual changes in pressure ^12, 50^.

## Clinical relevance of cerebral autoregulation monitoring

Despite the emerging availability of bedside physiology monitors, blood pressure targets remain mostly empirically chosen during critical care management. Efforts at preventing cerebral injury have emphasized the importance of maintaining a ‘normal blood pressure’ to ensure adequate perfusion of the brain. Hence, often medications are given to increase blood pressure in order to restore cerebral perfusion. However, as discussed before, the position and the shape of the autoregulation curve may have shifted away, with LLAs and ULAs which vary greatly and unpredictably ^14^ and an autoregulation plateau which may be very narrow ^15^. The clinical implication is that CBF might fluctuate as blood pressure fluctuates, exposing the patient to the risk of decreased cerebral flow and potential ischemia when blood pressure is relatively too low and conversely to the risk of increased cerebral flow and increased ICP when blood pressure is relatively too high. Since the individual limits of autoregulation are unknown, it is likely that empiric blood pressure management will result in patients having a MAP outside the autoregulatory range for at least part of the surgery or stay in intensive care. If autoregulation monitoring can individualize blood pressure targets, it could provide a more effective means for preventing ischemia–reperfusion injury than the current standard of care.

## Perspectives

With the sophistication of signal processing and online analysis software, the challenge lies in the successful integration of ‘optimal MAP’ and autoregulation monitoring into daily critical care management.The next steps will be to study the autoregulation in different clinical situations and to clarify whether incorporation into therapeutic management protocols leads to an improved cerebral outcome.

## Conclusions

Progress in perioperative and critical care will depend on moving away from broad assumptions and ‘one size fits all’ physiological targets. In the near future, real-time cerebral autoregulation monitoring may provide clinicians with the opportunity to individualize blood pressure targets and might direct critical care management to the individual patient’s need.
